# Age-Related Differences in the Anthropometric and Physical Fitness Characteristics of Young Soccer Players: A Cross-Sectional Study

**DOI:** 10.3390/children9050650

**Published:** 2022-05-01

**Authors:** Koulla Parpa, Marcos Michaelides

**Affiliations:** Sport and Exercise Science, UCLan University of Cyprus, Pyla 7080, Cyprus; mmichaelides@uclan.ac.uk

**Keywords:** chronological age, youth soccer, agility, jump performance, speed, handgrip strength

## Abstract

Considering that most professional academies seek to optimize the early detection and physical development of their younger players, the purpose of this study was to examine the anthropometric and physical fitness characteristics in a large cross-sectional sample of youth soccer players in Eastern Europe, starting from a very young age during their in-season period. Three hundred and thirteen soccer players (*n* = 313), grouped into eight age categories, participated in the study. On the basis of chronological age, the group categories were: 7 (*n* = 26), 8 (*n* = 41), 9 (*n* = 46), 10 (*n* = 48), 11 (*n* = 42), 12 (*n* = 47), 13 (*n* = 43), and 14 years old (*n* = 20). The players underwent an anthropometric evaluation, flexibility, handgrip strength, vertical jump performance, speed, and agility assessments. A one-way multivariate analysis of variance (MANOVA) indicated significant differences in the anthropometric and physical fitness variables based on chronological age (*F* = 13.40, *p* < 0.05, Wilk’s Λ = 0.08, partial η^2^ = 0.30). Concurrently, there were significant growth and physical fitness differences even in players born in the same chronological year. It is believed that the results have important practical implications, especially for those involved in youth soccer. Based on our results, coaches should contemplate speed and agility development in training sessions starting from a much younger age, as sprinting while changing directions has been considered an essential prerequisite in soccer.

## 1. Introduction

In contemporary soccer, players are required to possess a high level of physical fitness and technical skills to sustain the intensity of the games and ultimately support high performance, even in the case of youth players [1,2]. A soccer game imposes great physical and physiological demands where youth soccer players cover between 8 and 9 km per game, out of which 20–25% is performed as high- and very high-intensity running and sprinting [3]. Top-class soccer players perform more high-intensity running during a soccer game than semi- professional players [4]. Concurrently, soccer demands high levels of forceful and explosive movements such as jumps, sprints, and changes in direction [5]. These high-intensity events rely predominantly on the anaerobic energy system and require high strength generation. Although high-intensity demands are critical during a soccer game, the aerobic energy system’s predominance is evident during low- to moderate-intensity running demands. Thus, aerobic endurance increases the distance covered during a game, the number of sprints, and interactions with the ball [6]. In conclusion, these characteristics and performance indicators should be trained independently, especially in younger soccer players [7].

It is well established that understanding soccer games’ specific requirements can provide essential information for creating the most successful training programs to prepare young players for participating at an elite level and positively impact the talent identification process [5,8]. A plethora of studies have examined the anthropometric characteristics, aerobic repeated-sprint ability (RSA), and sprint performance of young soccer players and goalkeepers [7,9,10,11,12,13] as critical performance and talent indicators. In addition to the aforementioned indicators, researchers have examined critical technical aspects of kicking performance and ball velocity in soccer players, highlighting the need to improve the knee extension angular velocity for both the dominant and non-dominant legs [14,15], especially during the early stages of adolescence when bilateral imbalances become more evident [16]. Furthermore, investigators have examined the relationship between selected physical fitness parameters according to the playing positions in youth soccer players [17,18]. These results indicate that physical fitness and performance abilities between positions in young soccer players are different [17,18]. Additionally, anthropometry can discriminate physical capacities and soccer skills, providing a scientific rationale for player selection [17,18].

A battery of tests [19,20,21,22] including vertical and horizontal jumps, sprinting tests, agility, repeated sprint ability, handgrip strength, and aerobic capacity tests, is commonly used to distinguish between different age groups. Notably, these parameters become increasingly important, especially in young players where differences in growth and maturity result in performance disparities [11], opportunities, and, consequently, unequal competition presence according to their age and birth month [20,23,24]. In this regard, the importance of evaluating different skills, scaling children by age groups, and adding information to their anthropometric and performance profiles is highlighted.

Despite the large body of scientific evidence on anthropometric and physical fitness characteristics in young soccer players, knowledge regarding very young players is scarce, as most studies examine the aforementioned attributes from ages 10 and up. Therefore, considering that most professional academies seek to optimize the early detection and physical development of their young players, the purpose of this study was to examine the anthropometric and physical fitness variables in a large cross-sectional sample of youth soccer players from Eastern Europe, starting from a very young age during their in-season period.

## 2. Materials and Methods

### 2.1. Participants

Three hundred and thirteen young soccer players (*n* = 313), grouped into eight age categories, participated in the study. On the basis of chronological age, the group categories were: 7 (*n* = 26), 8 (*n* = 41), 9 (*n* = 46), 10 (*n* = 48), 11 (*n* = 42), 12 (*n* = 47), 13 (*n* = 43), and 14 years old (*n* = 20). All players engaged in formal training sessions (3–4 sessions/week, ~90 min/session) and participated in a nine-month competitive season, usually with one game played during the weekend over the entire training period. All of the procedures were conducted during the in-season period. All head coaches were UEFA-licensed coaches for the specific age groups. Parents or legal guardians provided written informed consent after receiving verbal and written information about the study’s procedures, associated risks, and benefits. The study was performed in accordance with the Declaration of Helsinki and was approved by the Ethics Committee of the University (reference number STEMH 541) and the National Committee on Bioethics [25]. The exclusion criteria included injuries or sickness resulting in losing two or more soccer games and/or training sessions two months prior to study initiation. Lastly, all the children had medical clearance to participate in soccer training and testing.

### 2.2. Measurements and Data Collection Procedures

The participants were advised to abstain from any activity the day before testing, and measurements were obtained between 14:00 and 17:00 during the players’ training hours. Specific familiarization sessions were performed before the evaluations as some tests were not part of the players’ training routines. Before the performance tests, the players performed a general 15-min warm-up, which consisted of submaximal running (low-to-medium intensity) and dynamic stretching. The warm-up also included coordination exercises such as running with lifted knees, heeling, and sidestepping. Furthermore, anthropometric measurements were recorded to determine the players’ height and body mass prior to the performance assessments. Stature and body mass were measured according to standard procedures [26] using a wall stadiometer (Leicester; Tanita, Tokyo, Japan) and a standard electronic scale, and recorded to the nearest 0.1 kg and 0.1 cm, respectively.

### 2.3. Sit and Reach Test

A custom sit and reach box (32.4 cm high and 53.3 cm long) with a 26 cm heel line mark was used to assess the flexibility of the lower back and hamstring muscles according to methods described by previous investigators [27]. The players placed the soles of their feet (no shoes) against the box with knees in full extension. They were instructed to lean forward with one hand on top of one another and palms facing downward. Fast and jerky movements were not allowed while they were leaning forward. The players performed three attempts, and the best trial recorded to the nearest centimeter was entered for statistical analysis.

### 2.4. Free Arm Countermovement Jump (CMJ)

Explosive strength power was assessed with the CMJ test. The vertical jump performance was evaluated using Optojump^TM^ photoelectric cells (Microgate, Bolzano, Italy), and the procedure was followed according to methods described by previous investigators [28]. Each player performed three countermovement jumps (CMJs) with the same break between jumps. The participants’ hands were placed on their waist, and swinging of the arms was not allowed. The highest of three valid jumps was included in the data analysis.

### 2.5. Handgrip Strength Test

A handgrip dynamometer (Takei Scientific Instruments Co., Ltd., Tokyo, Japan) was used to assess the maximum isometric strength of the forearm and hand muscles. The procedure was conducted according to the methods described by previous investigators [29].

### 2.6. Straight Sprint Tests

Sprint performance was evaluated using a maximal 30 m sprint (sprint times were recorded at 10, 20, and 30 m) according to the methods described by previous investigators [30]. The participants were instructed to perform a 30 m sprint with maximal effort, and speed was measured as the time elapsed in 10, 20, and 30 m. The elapsed time for each test was automatically recorded with precision using photocell gates (accuracy of 0.01 s; Brower Timing Systems, Salt Lake City, UT, USA) placed 0.4 m above the ground. The timing gate at 30 m recorded the total time taken to sprint 30 m. All players began with a standing start, with the front foot positioned 0.5 m from the first timing gate. The players performed two trials with a 2 min rest in between [30], and the best 30 m sprinting time was selected for the statistical analysis.

### 2.7. Agility *t*-Test

The *t*-Test is a simple agility test that involves forward, lateral, and backward running. For the *t*-Test, four cones were arranged into a T-shape according to the methods described by previous investigators [31]. The second cone was placed 9.14 m from the starting cone (photocell gates 2 m apart), and cones three and four were placed 4.57 m on either side of the second cone. The players had to sprint forward 9.14 m from the start line, right shuffle 4.57 m to the third cone, then shuffle 9.14 m left to the fourth cone, and 4.57 m back to the middle cone before they finally performed backward running to the start line. The trials were not considered if participants failed to touch a designated cone or failed to face forward at all times. Only one timing gate placed on the start–finish line was used for timing the *t*-test. Each test was repeated two times, and the best time was included in the data analysis.

### 2.8. Statistical Analysis

Statistical analysis was performed using SPSS (version. 16.0; SPSS Inc., Chicago, IL, USA). The results are presented as the means and standard deviations (SDs). The differences among the age groups in anthropometric and physical fitness characteristics were assessed using a one-way multivariate analysis of variance (MANOVA) followed by an LS means post-hoc analysis to identify which groups differed. The effect size was estimated with partial eta-squared (η^2^). The effect sizes were interpreted as follows: large (partial η^2^ ≥ 0.14), medium (partial η^2^ ≥ 0.06), and small (partial η^2^ ≥ 0.01 or more) [32]. Linear regressions of the dependent variables (anthropometric and performance data) vs. explanatory (chronological age) were visually inspected. The *R*^2^ values represented the proportion of variance for the anthropometric and physical fitness data explained by the chronological age. Statistical significance was set at a *p*-value of <0.05.

## 3. Results

**Figure 1:**
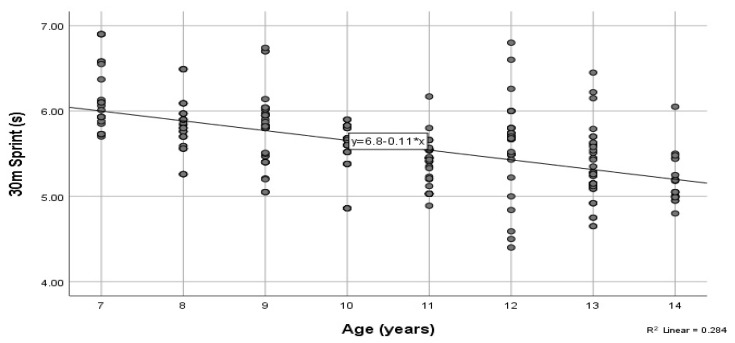
A scatter plot and the corresponding regression line for the relationship between the dependent variable, 30-m sprint (s), and the independent variable chronological age (years).

**Figure 2:**
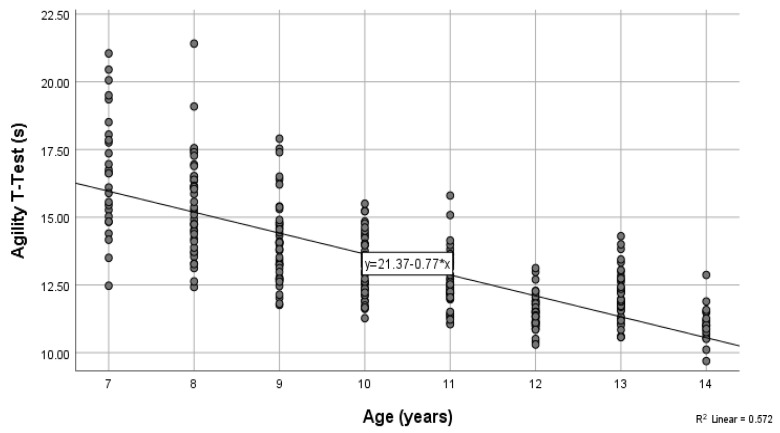
A scatter plot and the corresponding regression line for the relationship between the dependent variable, agility *t*-test (s), and the independent variable chronological age (years).

The anthropometric characteristics are presented in Table 1. The handgrip, flexibility, and vertical jump results are presented in Table 2, and the sprint and agility results are presented in Table 3. In addition, pairwise comparisons for height and body mass are presented in Table 4 and Table 5, respectively. The one-way analysis of variance MANOVA indicated statistically significant differences in the anthropometric and physical fitness variables based on chronological age (*F* = 13.40, *p* < 0.05, Wilk’s Λ = 0.08, partial η^2^ = 0.30). Further analysis indicated that age had a statistically significant effect on both height (*F*(7,305) = 90.53, *p* < 0.05, partial η^2^ = 0.68) and body mass (*F*(7,305) = 41.56, *p* < 0.05, partial η^2^ = 0.49). Furthermore, age had a statistically significant effect on handgrip strength (left hand, *F*(7,305) = 91.58, *p* < 0.05, partial η^2^ = 0.68; right hand, *F*(7,305) = 76.47, partial η^2^ = 0.64), flexibility (*F*(7,305) = 7.01, *p* < 0.05, partial η^2^ = 0.14), and vertical jump performance (*F*(7,305) = 34.03, *p* < 0.05, partial η^2^ = 0.44). Lastly, age had a statistically significant effect on 10 m (*F*(7,305) = 29.41, *p* < 0.05, partial η^2^ = 0.4), 20 m (*F*(7,305) = 21.93, *p* < 0.05, partial η^2^ = 0.34), and 30 m sprints (*F*(7,305) = 24.56, *p* < 0.05, partial η^2^ = 0.36), as well as agility *t*-Test performance (*F*(7,305) = 74.46, *p* < 0.05, partial η^2^ = 0.63). Based on the results, there was an increased rate of physical fitness in all variables based on age, except for the 12-year-old players who performed better than the 13-year-old players in handgrip strength, jump performance, flexibility, and the agility test (Table 2 and Table 3). The coefficient of determination for the relationship between height and age indicated that 62.7% of the variance in height was due to age. Furthermore, 44% of the variance in body mass was due to age (*r*^2^ = 0.439). The coefficients of determination for the right and left handgrip strength were 0.609 and 0.575, respectively. In addition, the coefficients of determination for flexibility and vertical jump indicated that only 9.6% of the variance in flexibility and 36% of the variance in countermovement vertical jump performance was due to age. Regarding the sprint test, 38.0%, 25.6%, and 28.4% of the proportions of variance in 10, 20, and 30 m sprint times (Figure 1), respectively, were explained by age. Lastly, 57.2% of the variance in the agility *t*-test was due to chronological age (Figure 2).

## 4. Discussion

**Figure 3:**
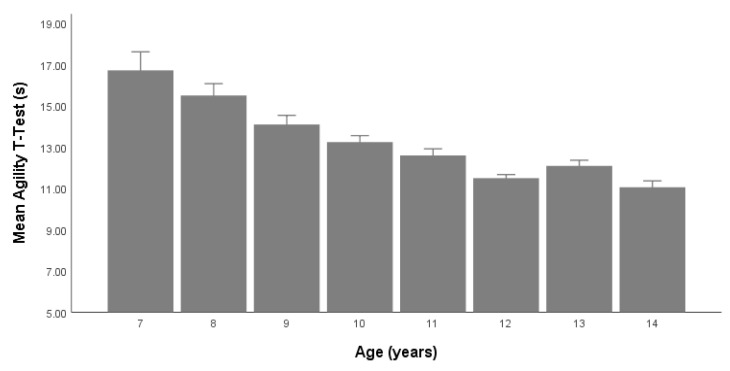
Agility *t*-Test (s) based on chronological age (years).

This study examined the anthropometric and physical fitness variables in a large cross-sectional sample of youth soccer players in Eastern Europe. We found that age had a statistically significant effect on height, body mass, handgrip strength, flexibility, vertical jump performance, sprint times, and agility *t*-Test performance. We also observed an increased rate of physical fitness in all variables based on age except for the 12-year-old players, who performed better than the 13-year-old players in handgrip strength, jump performance, flexibility, and the agility test (Table 2 and Table 3, Figure 3).

The results from this study generally support previous research, which suggested that age-related performance and anthropometry increase significantly with age [33]. However, to the best of our knowledge, this was the first study to examine a large sample of players (*n* = 313) starting from a very young age (over 50% of the children were younger than 10 years old). Considering that players are organized into specific teams within professional youth soccer based on their chronological age, the goal of this study was to assess the players based on chronological age and not biological maturation. The present results highlight the asynchronicity of this process, which aligns with previous investigations that demonstrated a highly individualized onset of the adolescent growth spurt and an asynchronous relationship with differing athletic performance indices in young soccer players [34].

Regarding the anthropometric measurements, the rate of development of body mass and height increased markedly at 11 and 12 years of age. Body mass increases were more evident at the age of 11, while height increases were more evident at 12, highlighting asynchronous development. Notably, within our sample, the onset of a growth spurt was apparent around the age of 12, with an average growth of around 7 cm/year. This finding aligns with previous studies that indicated an onset of the stature growth spurt at around 12 years old [35], whereas some researchers have demonstrated an even earlier onset in growth spurt at around 10.7 years old [33]. Although the 14-year-old players in our study were taller on average than other European players in Belgium [36], they had a similar stature to French professional soccer players between the ages of 14 and 16 years [37]. Lastly, our results indicated that 62.7% of the variance in height and 44% of the variance in body mass were due to age. The linear regressions indicated that there were significant growth differences even in players born in the same chronological year. Research has indicated that these differences may be attributed to the relative age effect [38] or differences in the biological maturity of the players [38]. Relevant to this is that players of the same chronological age may be ahead, on time, or behind their chronological age [39].

Of particular interest in the present study was that lower limb power, as identified by CMJ, did not follow the same trend as the anthropometric gains. The most significant increase in CMJ was identified at the age of 12, whereas 13- and 14-year-old players demonstrated lower CMJ results. These findings somewhat contrast with youth soccer studies demonstrating that overall jump ability improves with age [33]. Nevertheless, there may be several reasons for these discrepancies. First, we examined players from the age of 7–14 years, whereas other studies examined players from the age of 11–18 years. Therefore, from 14 to 18 years old, players undergo significant changes in growth and maturation, which play an essential role in neural and muscular adaptations and may explain the possible differences in CMJ performance across the age groups [23]. In a study by Williams et al., (2011) [33] where u11–u16 players were examined, CMJ improved with age, but there was no significant difference between the players in the U12 and U13 teams. Therefore, the age-related jump height differences mainly referred to older players (U14, U15, U16). Furthermore, lower limb power does not require any technical skills or multi-joint coordination, which may explain why 12-year-olds performed better on CMJs than 13- and 14-year-old players. Lastly, the observation that the 12-year-old players in this study were more physically developed than the 13-year-olds may partly account for our finding. Fourteen-year-old players demonstrated significantly greater values in handgrip strength, whereas no differences were identified among the 12- and 13-year-olds. The handgrip strength data revealed that the development of upper body strength also follows an asynchronous process. Therefore, even though handgrip strength was generally observed to increase based on chronological age, those increases were highly individualized and not linear based on age. For example, the 10-year-old players demonstrated significantly greater mean handgrip strength than the 11-year-old players.

The importance of sprinting or the ability to perform high-speed running is highlighted considering high-intensity running, which is evident during soccer games [40,41]. Additionally, a longitudinal study on Danish soccer players indicated that the best talent indicator for predicting selection for professional academies was the 30 m sprint speed [42]. Our results revealed that sprint times improved with age for the 10 m run, whereas 11-year-old players performed best in the 20 m run. However, the 14-year-old players produced faster sprint times during the 30 m sprint test and agility *t*-Test. Regarding the agility *t*-test, older players performed significantly better than the rest of the age groups, which may be explained by their more efficient movement pattern; therefore, they had a greater ability to change direction than the younger players. Overall, the players in our study produced slower sprint times in the 10 and 20 m tests compared with Scottish players [43], whereas the results of the 13- and 14-year-old players aligned with the results of Portuguese soccer players [22].

## 5. Conclusions

To the best of our knowledge, this is the first study in Eastern Europe to include soccer players at the age of seven. Our research revealed that age had a statistically significant effect on height, body mass, handgrip strength, flexibility, vertical jump performance, sprint times, and agility *t*-test performance. Concurrently, there were significant growth and performance differences, even in players born in the same chronological year. Our results suggest that academies should conduct longitudinal monitoring to update the players’ anthropometric and physical fitness status. At the same time, soccer academies should implement age-appropriate and progressive training methods to optimize performance. Although success in soccer does not rely solely on physical fitness, these results can be combined with technical and tactical abilities to ensure high levels of success. Furthermore, we suggest that coaches contemplate speed and agility development in the period of training sessions starting from a much younger age as young players performed significantly worse on those. Although the decision for the players’ exercise programs is multifactorial, it is evident that players with the same chronological age have significant differences in anthropometric and physical fitness variables; thus, programs should be designed along a continuum, beginning with the safest and gradually progressing to more challenging exercise routines. Finally, coaches should emphasize agility tasks when developing exercise routines for youth soccer players, even at the age of seven, as the ability to change the body’s position and direction has been considered an essential prerequisite in soccer.

## 6. Limitations

A major limitation of the study is that it did not consider the relative age effect, which is the difference in age between children born in the same year.

Therefore, future studies should be encouraged to split the year into quartiles before examining young players’ chronological and physical fitness parameters.

## Figures and Tables

**Table 1:** Anthropometric characteristics.

Age Category (Years)		Height (cm)	95% CI	Body Mass (kg)	95% CI
	n	Mean ± SD	Lower Bound Upper Bound	Mean ± SD	Lower Bound Upper Bound
7	26	127.62 ± 6.11	125.15–130.08	27.04 ± 4.60	25.19–28.90
8	41	133.45 ± 6.16	131.51–135.40	30.06 ± 5.22	28.41–31.71
9	46	137.61 ± 4.85	136.17–139.05	35.47 ± 5.71	33.78–37.17
10	48	142.07 ± 5.93	140.35–143.80	37.63 ± 8.18	35.25–40.00
11	42	148.69 ± 6.30	146.73–150.65	42.28 ± 7.54	39.93–44.63
12	47	155.80 ± 9.44	152.25–157.79	46.79 ± 10.37	43.74–49.83
13	43	150.80 ± 7.16	148.60–153.00	42.99 ± 6.79	40.90–45.08
14	20	163.64 ± 8.35	159.73–167.54	54.65 ± 9.94	49.99–59.30

Note: CI: Confidence interval.

**Table 2:** Handgrip, flexibility, and counter-movement jump (CMJ).

Age Category (Years)		Left Handgrip (kg)	Right Handgrip (kg)	CMJ (cm)	Flexibility (cm)
	n	Mean ± SD	Mean ± SD	Mean ± SD	Mean ± SD
7	26	10.62 ± 2.20	11.17 ± 2.05	18.91 ± 3.58	23.27 ± 6.40
8	41	13.17 ± 2.51	13.12 ± 2.89	18.78 ± 3.52	23.23 ± 6.25
9	46	14.76 ± 2.27	14.83 ± 2.76	21.96 ± 4.07	26.20 ± 5.89
10	48	18.96 ± 2.75	19.27 ± 3.35	24.20 ± 4.23	25.29 ± 5.21
11	42	16.70 ± 3.40	17.20 ± 4.57	25.85 ± 3.85	24.67 ± 6.50
12	47	24.86 ± 5.40	25.89 ± 6.07	29.86 ± 5.11	29.15 ± 7.38
13	43	24.45 ± 5.17	25.29 ± 6.01	28.17 ± 5.28	27.49 ± 6.12
14	20	30.33 ± 5.37	30.80 ± 5.91	26.50 ± 4.80	32.25 ± 6.45

**Table 3:** Sprint and agility (*t*-Test).

Age Category (Years)		10 m (s)	20 m (s)	30 m (s)	Agility *t*-test (s)
	n	Mean ± SD	Mean ± SD	Mean ± SD	Mean ± SD
7	26	2.52 ± 0.23	4.22 ± 0.23	6.19 ± 0.41	16.71 ± 2.22
8	41	2.51 ± 0.21	4.01 ± 0.20	5.83 ± 0.31	15.49 ± 1.81
9	46	2.39 ± 0.22	4.02 ± 0.26	5.78 ± 0.39	14.10 ± 1.49
10	48	2.30 ± 0.19	3.75 ± 0.38	5.54 ± 0.30	13.24 ± 1.07
11	42	2.23 ± 0.11	3.51 ± 0.41	5.40 ± 0.25	12.59 ± 1.03
12	47	2.18 ± 0.12	3.72 ± 0.39	5.63 ± 0.44	11.49 ± 0.60
13	43	2.14 ± 0.12	3.54 ± 0.34	5.30 ± 0.39	12.08 ± 0.89
14	20	2.12 ± 0.12	3.58 ± 0.21	5.18 ± 0.29	11.05 ± 0.66

**Table 4:** Pairwise comparisons for Height.

Age Category (Years)	Age Category (Years)	Mean Difference For Height	Std. Error	95% CI for Difference Lower Bound Upper Bound
7	8	−5.84 *	1.72	−9.22–(−2.45)
	9	−9.99 *	1.68	−13.3–(−6.68)
	10	−14.46 *	1.67	−17.75–(−11.17)
	11	−21.08 *	1.71	−24.45–(−17.70)
	12	−27.41 *	1.67	−30.71–(−24.10)
	13	−23.19 *	1.71	−26.54–(−19.83)
	14	−36.02 *	2.04	−40.03–(−32.00)
8	9	−4.16 *	1.47	−7.06–(−1.26)
	10	−8.62 *	1.46	−11.49–(−5.75)
	11	−15.24 *	1.51	−18.20–(−12.27)
	12	−21.57 *	1.47	−24.46–(−18.69)
	12	−17.35 *	1.50	−20.30–(−14.41)
	14	−30.18 *	1.87	−33.86–(−26.50)
9	10	−4.46 *	1.42	−7.25–(−1.68)
	11	−11.08 *	1.46	−13.96–(−8.20)
	12	−17.41 *	1.42	−20.21–(−14.61)
	13	−13.20 *	1.46	−16.06–(−10.33)
	14	−26.03 *	1.84	−29.64–(−22.41)
10	11	−6.62 *	1.45	−9.47–(−3.76)
	12	−12.95 *	1.41	−15.72–(−10.18)
	13	−8.73 *	1.44	−11.57–(−5.90)
	14	−21.56 *	1.92	−25.16–(−17.97)
11	12	−6.33 *	1.45	−9.2–(−3.46)
	13	−2.11	1.49	−5.04–(0.82)
	14	−14.94 *	1.86	−18.61–(−11.28)
12	13	4.22 *	1.45	1.37–(7.07)
	14	−8.61 *	1.83	−12.22–(−5.01)
13	14	−12.83 *	1.86	−16.49–(−9.17)

* *p* < 0.05.

**Table 5:** Pairwise comparisons for Body mass.

Age Category (Years)	Age Category (Years)	Mean Difference For Body Mass	Std. Error	95% CI for Difference Lower Bound Upper Bound
7	8	−3.01	1.89	−8.97–(2.94)
	9	−8.43 *	1.85	−14.26–(−2.60)
	10	−10.59 *	1.84	−16.37–(−4.80)
	11	−15.24 *	1.88	−21.16–(−9.31)
	12	−19.74 *	1.84	−25.55–(−13.94)
	13	−15.95 *	1.87	−21.85–(−10.05)
	14	−27.60 *	2.24	−34.67–(−20.54)
8	9	−5.42 *	1.62	−10.52–(−0.31)
	10	−7.57 *	1.60	−12.62–(−2.52)
	11	−12.22 *	1.65	−17.44–(−7.01)
	12	−16.73 *	1.61	−21.81–(−11.66)
	13	−12.94 *	1.65	−18.12–(−7.75)
	14	−24.59 *	2.06	−31.07–(−18.11)
9	10	−2.16	1.55	−7.02–(2.74)
	11	−6.81 *	1.61	−11.88–(−1.74)
	12	−11.32 *	1.56	−16.24–(−6.39)
	13	−7.52 *	1.60	−12.56–(−2.48)
	14	−19.17 *	2.02	−25.53–(−12.81)
10	11	−4.65	1.59	−9.67–(0.37)
	12	−9.16 *	1.55	−14.03–(−4.28)
	13	−5.36 *	1.58	−10.35–(−0.38)
	14	−17.02 *	2.01	−23.34–(−10.69)
11	12	−4.51	1.60	−9.55–(0.53)
	13	−0.71	1.63	−5.87–(4.44)
	14	−12.37 *	2.05	−18.82–(−5.91)
12	13	3.79	1.59	−1.22–(8.81)
	14	−7.86 *	2.01	−14.20–(−1.52)
13	14	−11.65 *	2.04	−18.08–(−5.22)

* *p* < 0.05.

## Data Availability

Data can be obtained by contacting the lead author K.P.
